# Flow through reactors for organic chemistry: directly electrically heated tubular mini reactors as an enabling technology for organic synthesis

**DOI:** 10.3762/bjoc.5.70

**Published:** 2009-11-30

**Authors:** Ulrich Kunz, Thomas Turek

**Affiliations:** 1 Institute of Chemical Process Engineering, Clausthal University of Technology, Leibnizstr. 17, D-38678 Clausthal-Zellerfeld (Germany)

**Keywords:** direct electric heating, flow reactors, micro reactors, organic chemistry, reaction kinetics

## Abstract

Until recently traditional heating in organic chemistry has been done with oil heating baths or using electric heat exchangers. With the advent of microwave equipment, heating by microwaves was rapidly introduced as standard method in organic chemistry laboratories, mainly because of the convenient possibility to operate at high temperature accompanied by accelerated reaction rates. In the present contribution we discuss the method of heating small, continuously operated reactors by passing electric current directly through the reactor wall as an enabling technology in organic chemistry. The benefit of this method is that the heat is generated directly inside the reactor wall. By this means high heating rates comparable to microwave ovens can be reached but at much lower cost for the equipment. A tool for the comparison of microwave heating and traditional heating is provided. As an example kinetic data for the acid catalyzed hydrolysis of methyl formate were measured using this heating concept. The reaction is not only a suitable model but also one of industrial importance since this is the main production process for formic acid.

## Introduction

Continuously operated small reactors for organic synthesis have gained much interest during the last years. Micro reaction technology is now well known and applied in many laboratories [1–2], with many examples of its application in organic synthesis [3–6]. One aspect still preventing widespread use is the high cost of most microreactors and the peripheral equipment which is necessary for heat and flow control. Most of these reactors are indirectly heated, by fluid-supplied heating jackets or by electric heating cartridges and jackets. Beside oil baths and electric heaters microwave ovens are now widely present in the organic chemist’s laboratory [7]. In many cases shorter reaction times have been reported compared to the traditional heating methods. The question of a specific microwave effect is under current discussion, but for many chemical reactions it is evident that microwave heating is just another way transferring heat into the reaction mixture [8–9]. The constructions of closed vessels used as reactors in microwave ovens allow operation under pressure. Thereby the reaction temperature obtainable can be much higher than in traditionally heated open vessels and beakers, leading to shorter reaction times. This fact, in combination with the very convenient use of microwave ovens comparable to kitchen equipment and safe operation in a closed cabinet, are the reasons for the success of this technology.

The high cost and dimensions in the sub millimeter range led us to the question: what other methods are available to heat small flow through reactors conveniently and rapid at low cost?

## Results and Discussion

### Heating concept

In addition to microwaves, heating by other electromagnetic fields was recently introduced into organic synthesis. Magnetic particles were heated by the interaction with an electromagnetic field in the frequency range of long radio waves (< 100 kHz) [10]. Both traditional and these novel methods have disadvantages. The disadvantages of the traditional heating methods are:

- Oil baths have large volumes of oil, are bulky and heat up rather slowly. The synthesis flasks have to be cleaned from adhering oil.
- Electric heating baskets are electrically isolated for safety reasons, so the heat transfer to the glass flask is rather limited, making these devices slow in operation. Due to the electric insulation the heating wire inside may have a much higher temperature than the surface of the heated flask. This may be an ignition source for organic solvent vapors.

Heating with electromagnetic devices like microwave ovens or inductive heaters has other disadvantages:

- Only a small part of the energy taken from the AC power line is transferred as heat into the chemical reactor.
- The equipment is expensive because it requires well-controlled electromagnetic field generation.
- Cheap thermocouples cannot be used as sensors for temperature measurement and control.
- Temperature sensors cannot always be fixed directly into the reaction mixture because of limited chemical or mechanical stability.
- Operation under high pressure is limited by the properties of the construction materials for the reactors which have to be transparent for electromagnetic waves.
- Homogeneous temperature distribution in the synthesis flasks is not always ensured since not all of these devices have a stirrer or mixer.
- Penetration depth of electromagnetic waves is limited.
- Shielding of the heating chamber is required to prevent electromagnetic waves entering the environment.

In this paper we provide an example of a much simpler method to heat small flow reactors. From our point of view the easiest way to transfer heat is to heat the reactor wall directly by passing electric current through the reactor wall itself. By resistive heating of the wall the heat transfer is facilitated because heat transfer has only to occur from the reactor wall to the reaction mixture. Additional heat transfer from an outer heating source to the reactor wall is avoided. Moreover the heat is transferred very uniformly across the whole surface area of the reactor, thus enlarging the effective heat transfer surface area, avoiding hot spots and preventing overheating of sensitive reactants. The response of the reactor to changes in the heating current is fast due to the low mass of the heating resistor, which is just the reactor wall.

This heating method is not new, it is well known to every plumber to melt the ice in frozen water pipes just by connecting the ends of the pipe with a welding transformer and let the electric current generate heat. Surprisingly, this heating method is seldom used in chemistry or other technical applications. One example is the injector liner in mass spectrometers or gas chromatograph mass spectrometers. This injector tube is a capillary with a few cm length which is heated by an electric current [11]. Another application is the preheating of liquid in cars e.g. for preheating the motor cooling fluid [12]. But in the device described the resistor wire is coiled onto a tube which is used for conducting the fluid. The tube itself is not used as the heating element. Heating micro reactors made of finely structured plates by electric current is also mentioned in the literature, [13–14] but to our knowledge resistive direct heating of the reactor wall is not yet used widely as a method to heat chemical reactors. A reason for this might be that high currents at low voltages are needed. This is necessary because the resistance of metal tubes or sheets used for reactor construction is low (in the milliohm range). Usually this is encountered with big losses in the switching semiconductors and in the wires used for connecting the electric circuit. Only recently lightweight switching power supplies with high load currents and high efficiency became available. Today the switching of high currents in the range of several 10 to 100 A without big losses is possible with metal oxide field effect transistors (MOSFET) having very low saturation voltages and on-resistances in the milliohm range. Based on the availability of modern electronic components the reactor concept of resistive heating is still limited to small reactors, that means diameters in the range of several millimeters and lengths to several 0.1 m up to 1–2 m are possible. From cost aspects micro reactors made of thin metal plates with channel dimensions in the sub millimeter range in combination with their sensitivity to mechanical failure like plugging, are not really an enabling technology for organic chemistry. Thus, the use of standard metal tubes in the aforementioned dimension range appears to be much more attractive. These dimensions fit the trend to use micro reactors or mini reactors for continuous chemical production perfectly.

Recently we introduced this concept as an attractive method for organic reactions with preliminary measurements [15]. In this paper we give a detailed description of the system and possible applications in organic synthesis.

### Experimental set up

**Figure 1:**
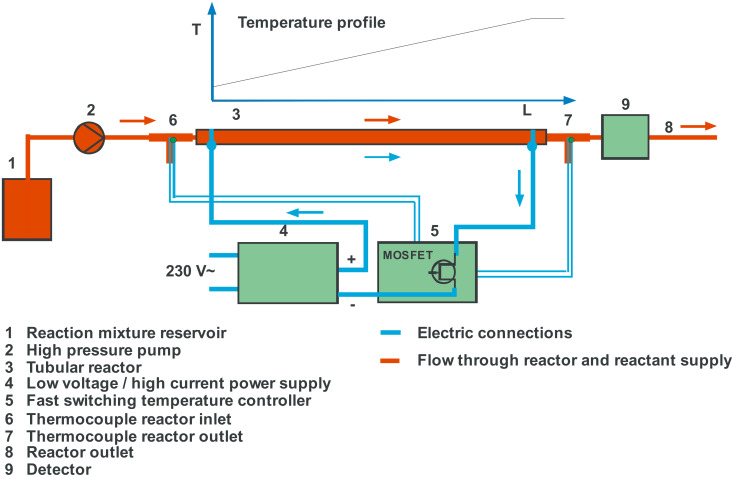
Experimental setup for heating tubular flow reactors by passing electric current directly through the reactor wall. The characteristic linear temperature profile along the reactor length is also given.

For the experimental evaluation of this heating concept we prepared different reactors which were heated by an electric current. The reactors were investigated in an apparatus depicted in Figure 1. The reaction mixture from reservoir (1) was pumped by a pump (2) to the inlet of the reactor (3). The reactors were made from 1/8″ or 1/4″ standard stainless steel tubing. The electric heating current was fed to the reactor by soldered solid 4 mm diameter copper wires which were also used to fix the reactor on a mounting base plate. The copper wires were connected with plugs for easy exchange of the different reactors. Heating current was delivered by a low voltage/high current power (5 V, 120 A) supply (4) controlled by a fast acting temperature controller (5). This temperature controller included a switching pulse width modulator with a frequency of approximately 30 Hz to ensure fast response of the low mass heater [16]. The reactor outlet temperature was regulated taking a thermocouple (7) as a measure. This thermocouple is located in the middle of the liquid stream leaving the reactor. Thereby the temperature of the liquid is adjusted, not the wall temperature of the reactor. To allow operation at high pressure inside the tube the outlet was attached with a capillary to introduce a pressure drop. Conversion was measured with a detector (9) connected directly behind the capillary before the reactor outlet (8). All electric connections within the heater supply lines were made of 10 mm^2^ flexible insulated copper wire to minimize resistive losses in the conductors.

## Experimental

### Power efficiency and dynamic behavior of the system

For these purposes a reactor made of 1/8″ stainless steel tubing (3.18 mm × 0.56 mm with a length of 50 cm) was used. The chosen material was 1.4404 stainless steel. For the reactor design it is necessary to know the specific electric resistance. A value of 0.724 Ω mm^2^ m^−1^ was measured at 25 °C. Before the reactor was used for reaction experiments the electric power consumption was estimated. For this purpose a flow of 10 mL water per min was pumped through the tube and the heating power was set to 100%. The water flow was used to cool the tube to prevent overheating. This mode of operation can only be used for a few seconds, because finally the water will evaporate, but this time is long enough to measure the characteristic data. The measured electric current flowing through the tube was 56.66 A, while a voltage at the tube of 4.45 V was determined. This gives a power uptake of the tube of 252 W. All this power is transferred into heat. The voltage drop of 0.55 V is mainly caused by the voltage drop of the power MOSFET switch which is used to actuate the heating current. The voltage drop of the copper cables can be neglected. The loss of the power switch is 31 W which is approximately 10% of the power taken from the power supply, thus the power supply has an efficiency of 90%. This leads to a power consumption from the 230 V ~ AC line of about 314 W to deliver the power of 283 W for the heated tube and the 31 W for the power switch. The power consumption of the temperature controller is about 1 W and can be neglected. Thus the overall efficiency is roughly 80%. To give an impression for a reachable temperature increase: with a power of 252 W the temperature of a water flow of 50 mL min^−1^ can be increased by 72 °C. This is more than sufficient for the flow regime of the planned experiments with a flow rate of the reaction mixture between 1 and 10 mL min^−1^.

Subsequently, the dynamic behavior with respect to temperature changes was studied. With the pump, water was fed at different flow rates between 1 and 10 mL min^−1^ through the reactor. Times were measured until the reactor outlet temperature had reached a steady value. Outlet temperatures were chosen in the range of 30 to 90 °C. The desired reactor outlet temperature was reached in about a few seconds almost irrespective of the flow rate. The heating time was hard to measure accurately due to the rapid heating. It is evident that with the high heating power and the small flow rates very short response times can be achieved to make a reaction mixture reach a desired temperature. Due to the linear temperature profile which is caused by the interaction of the constant volumetric heat generation inside a volume element of the reactor wall and the flowing liquid, a gentle rise of the temperature without overheating can be reached. Although gentle, the heating rate is fast, comparable with heating rates of microwave ovens.

The data given above demonstrate the dynamic behavior of the heating power. Also important for the performance of a chemical reaction is the residence time of the reaction mixture in the reactor. With the tube dimensions given above the residence time is 10 s at a flow rate of 10 mL min^−1^. That means that a reaction mixture can be heated within 10 s to a desired outlet temperature. It should be noted that the linear temperature profile for a set outlet temperature is constant, independent of the flow rate within the above given flow regime. If the tube is filled with inert solid material the free volume can be reduced by about a factor of two, which means that the residence time at the given flow rate of 10 mL min^−1^ is about 5 s. This approach is cheaper than to use high priced capillary tubing and is easier to handle in case of blockage. Such a high heating rate resulting in a linear temperature profile cannot be reached by traditional heating methods in an easy way.

### Kinetic measurements

**Scheme 1:**
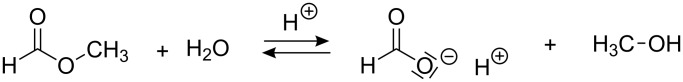
Acid catalyzed hydrolysis of methyl formate.

The applicability for kinetic measurements is demonstrated using a simple organic model reaction. We chose the hydrolysis of methyl formate with an ion exchange resin as an acidic heterogeneous catalyst. We chose a polymer catalyst because polymers are well defined materials which can be functionalized easily with many active groups. This leads to a wide variety of materials, including composites, suitable as catalysts or for anchoring active sites leading to a large number of composites [17–24]. The reaction scheme for the model reaction is given in Scheme 1.

This reaction was chosen for several reasons:

- Due to the low boiling point of the ester (32 °C) kinetic measurements in traditional equipment are limited to an upper temperature of about 25 °C, otherwise the vapor pressure would lead to loss of reactant in an open system. Measurements at temperatures higher than 32 °C are not possible at all.
- The progress of the reaction can be measured easily online by determining the electrical conductivity of the reaction mixture leaving the reactor. Electrical conductivity is directly proportional to the formation of formic acid and thus proportional to conversion. As a measure for conversion the conductivity of the reaction mixture at the reactor outlet was used.
- The reaction follows first order kinetics (within the concentrations we have chosen), which facilitates the determination of kinetic parameters.
- The reaction is of industrial importance. It is the main process for production of formic acid.

For the kinetic measurements two directly electrically heated and independently temperature regulated tubes in series were used. The same power supply delivers enough current for both tubes. The first tube had a diameter of 1/8″ and a length of 50 cm. This tube was used as a non catalytic preheater. The second tube was 100 cm in length and had a diameter of 1/4″. It was filled with an ion exchange resin catalyst (Amberlyst 70, mean particle diameter 0.8–1 mm, ion exchange capacity 0.7 mmol mL^−1^). The measurements were done by continuously feeding a mixture of 940 mL water, 490 mL methanol and 123 mL methyl formate to the first tube. This corresponds to an ester concentration of 1.275 mol L^−1^. This concentration was chosen because in many industrial organic syntheses the concentration of the reactant is about 1 mol L^−1^ [25]. During all measurements the outlet temperature of the preheater was set to the desired reaction temperature value. The outlet temperature of the catalytic reactor was set to the same temperature to allow isothermal operation. In most experiments the reaction temperature was set at much higher values than the boiling temperature of the ester at ambient pressure. This could be done since the reactor outlet was connected with an adjustable flow control valve and a capillary which were used to keep the reaction mixture under pressure in a liquid state. Even at a reaction temperature of 80 °C the ester does not vaporize because the mixture is under pressure. Most of the heating was done by the preheater: the catalytic reactor only needed to compensate heat loss to the environment. This concept, using a preheater, which contained no catalyst, and a flat, almost isothermal temperature profile along the catalytic reactor, prevents thermal damage from the polymer resin catalyst. In addition to this the temperature difference between the reactor wall and the reaction mixture was measured. This was done by thermocouples inside the reactor and with an infrared camera observing the temperature profile at the outer surface of the reactor. Both independent measurements as well as mathematical modeling of the temperature profiles reveal that the temperature difference between inside and outside of the preheater tube are much below 10 °C [26]. The catalytic reactor was operated at a much lower temperature gradient. The determined difference is not a problem for the resin catalyst. It is a feature of this reactor heating concept that the heating is fast with high power, but gentle because the heat is introduced into the reaction mixture along the whole reactor length with a linear rising temperature profile. Even sensitive compounds in a homogeneous reaction mixture will not be damaged because the temperature gradients are low.

**Figure 2:**
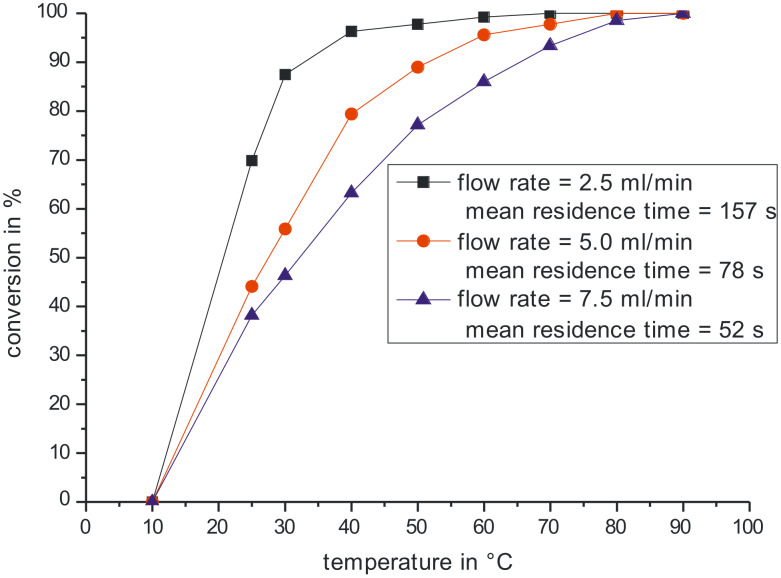
Conversion as a function of temperature for 3 different residence times.

Before performing the experiments the conversion at low temperature was estimated in a batch experiment. At a temperature of 10 °C negligible conversion was observed for several hours, so this temperature was set to approximate zero conversion. Then for three different flow rates conversion in dependence of the reactor temperature was measured. The results of these measurements are depicted in Figure 2. It can be seen that complete conversion can be reached at approximately 80 °C and a residence time of about one minute. The measurements were used to determine reaction rate constants and the activation energy.

As the reaction is first order the reaction rate constants can easily be calculated by

**Figure 3:**
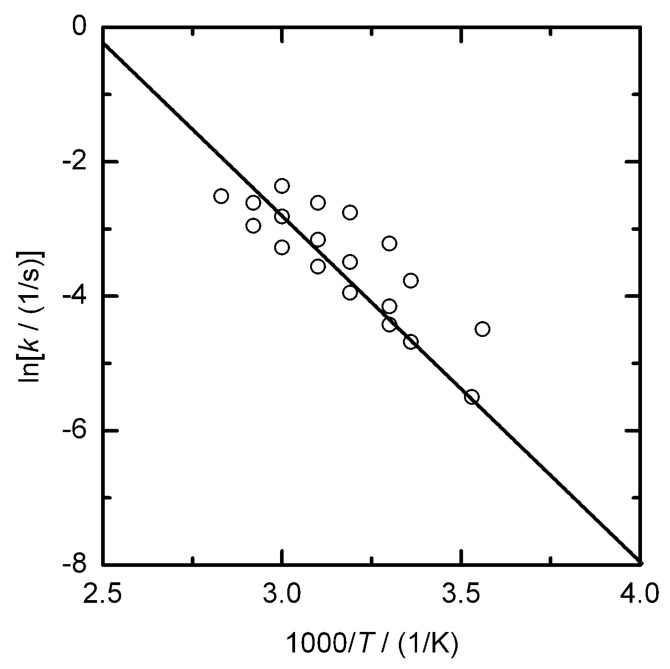
Arrhenius plot for the measured rate constants.

[1]
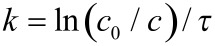


where τ is the mean residence time during the experimental run. *c*_0_ is the initial concentration of the ester and *c* is the current concentration, both in mol L^−1^. From the experiments 19 values for the reaction rate constant *k* could be calculated which were used to prepare an Arrhenius plot. This plot (Figure 3) was used to estimate the activation energy and the pre-exponential factor.

To some extent the values for k scatter. This could be based on the conductivity cell which was used for measuring the conversion as it had no temperature control. Measured values showed some influence on fluid temperature. From the Arrhenius plot an activation energy of 43.0 kJ mol^−1^ and a pre exponential factor of 3.27×10^5^ s^−1^ was determined. The directly heated tubular reactors have low mass and act fast to new temperature settings. After a waiting time of 5 to 6 residence times (which corresponds to a few minutes) the new temperature values have stabilized and measurements can be taken from the conductivity cell. The system demonstrates that directly heated small reactors can be used very efficiently in a convenient way. The chosen model reaction is only slightly exothermic. With reactions of much higher heat production the temperature profile at high conversion will be no longer linear. But this is not a big disadvantage, because it is well known to those skilled in the art that kinetic measurements can be done easily at low conversion. In such a case the reactor is operated in a differential mode to measure reaction rates at nearly constant feed concentrations. The simplest ways to do this are to lower the residence time in the continuously operated tubular reactor by using a higher flow rate or the dilution of the reaction mixture by a solvent.

## Conclusion and Outlook

A simple system was used for heating continuously operated small tubular reactors by passing electric current directly through the reactor wall. All components are standard laboratory equipment with much lower cost compared to heating devices using electromagnetic fields or finely structured micro reactors. The voltages necessary for heating are low and all parts can be touched without the risk of electric shock. Additional insulation is unnecessary either for electric safety or for heat management, leading to a system where all tube connectors can be reached easily. This makes the device handy for facilitating changes of the reactors which could be necessary for the adjustment of residence time or catalyst exchange. Even contamination by residues of previous reactions is not a problem. The reactors are cheap standard tubes, so they can be discarded and replaced by new ones. With the design guidelines given here it is easy to set up a continuously operated mini reactor for chemical reactions using readily available laboratory equipment. In this sense it is an enabling technology allowing the use of continuous reactors at low cost, enlarging the portfolio of enabling technologies for organic synthesis [27]. Performing a simple organic reaction demonstrates that the concept of directly electrically heated tubular reactors is convenient in use and gives high heating rates. The pressure resistant system allows operation well beyond the boiling temperatures of most solvents. Due to the use of low diameter stainless steel tubing, pressures up to even more than 100 bar are feasible. The applicability for kinetic measurements at temperatures above the normal boiling point was shown for the heterogeneously catalyzed hydrolysis of methyl formate. As an example the activation energy and rate constants for an industrial ion exchange resin catalyst were determined. The system gives the opportunity to adjust accurately high heating rates with traditional resistive electric heating. This feature makes the device a tool well suited to compare the influence of heating rates on chemical reactions. Further studies will focus on the comparison of heating within microwave ovens and this method as well as the application in organic flow synthesis.

## Supporting Information

File 1 Photograph of the current connector.

File 2 Photograph of the mounted reactor current connector.

File 3 Photograph of the preheater connection.

File 4 Photograph of the reactor setup.

File 5 Photograph of the thermocouple connection.

File 6 Photograph of different tubular reactors.
