# Supplementary figures and images for: Flow through reactors for organic chemistry: directly electrically heated tubular mini reactors as an enabling technology for organic synthesis
Source: Beilstein J Org Chem. 2009 Nov 30;5:70. doi: 10.3762/bjoc.5.70 (PMC2839532; doi:10.3762/bjoc.5.70)

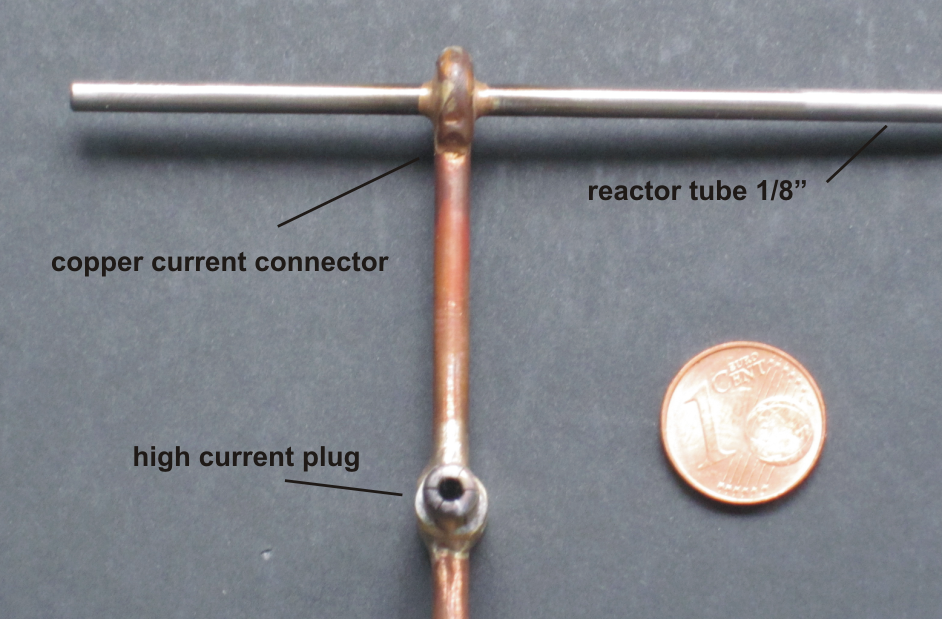

Supplement: File 1 — Photograph of the current connector. [file Beilstein_J_Org_Chem-05-70-s001.tif]

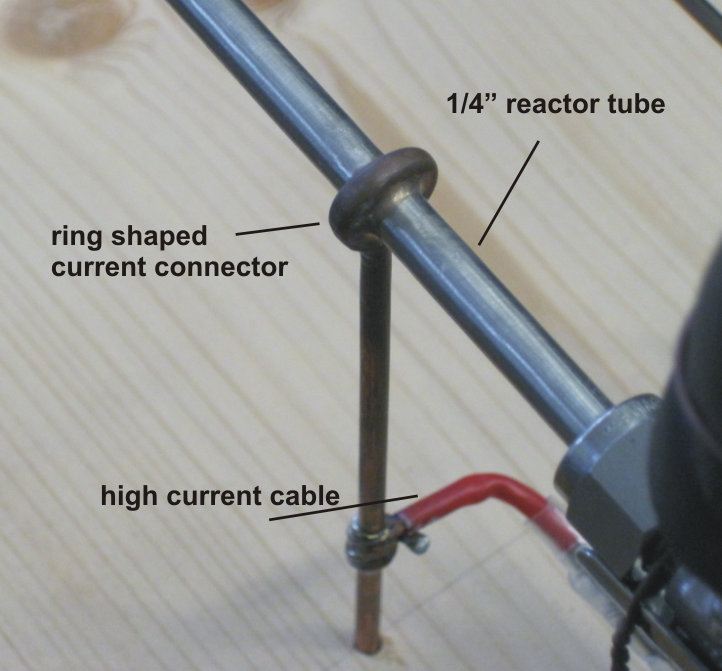

Supplement: File 2 — Photograph of the mounted reactor current connector. [file Beilstein_J_Org_Chem-05-70-s002.tif]

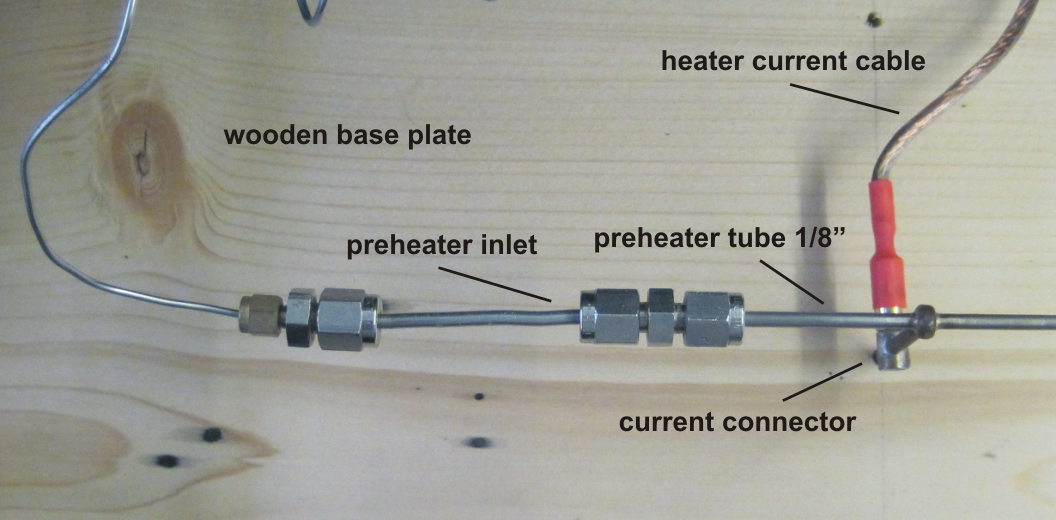

Supplement: File 3 — Photograph of the preheater connection. [file Beilstein_J_Org_Chem-05-70-s003.tif]

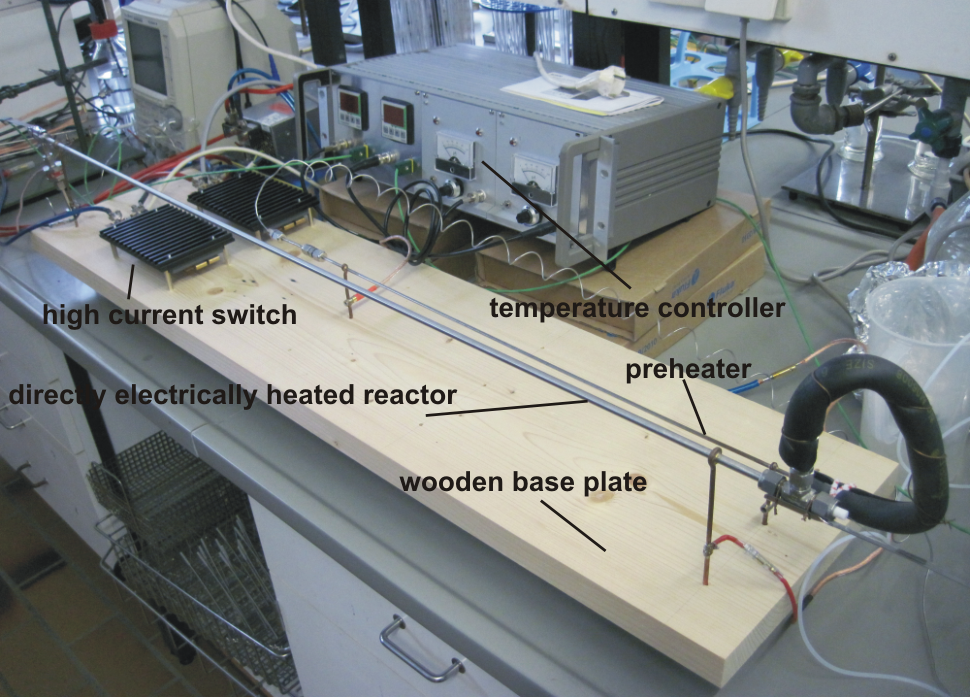

Supplement: File 4 — Photograph of the reactor setup. [file Beilstein_J_Org_Chem-05-70-s004.tif]

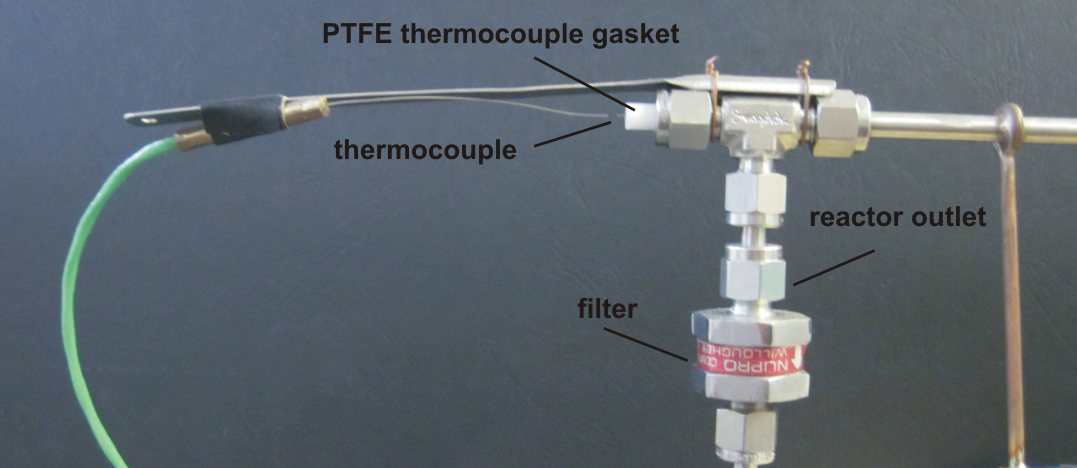

Supplement: File 5 — Photograph of the thermocouple connection. [file Beilstein_J_Org_Chem-05-70-s005.tif]

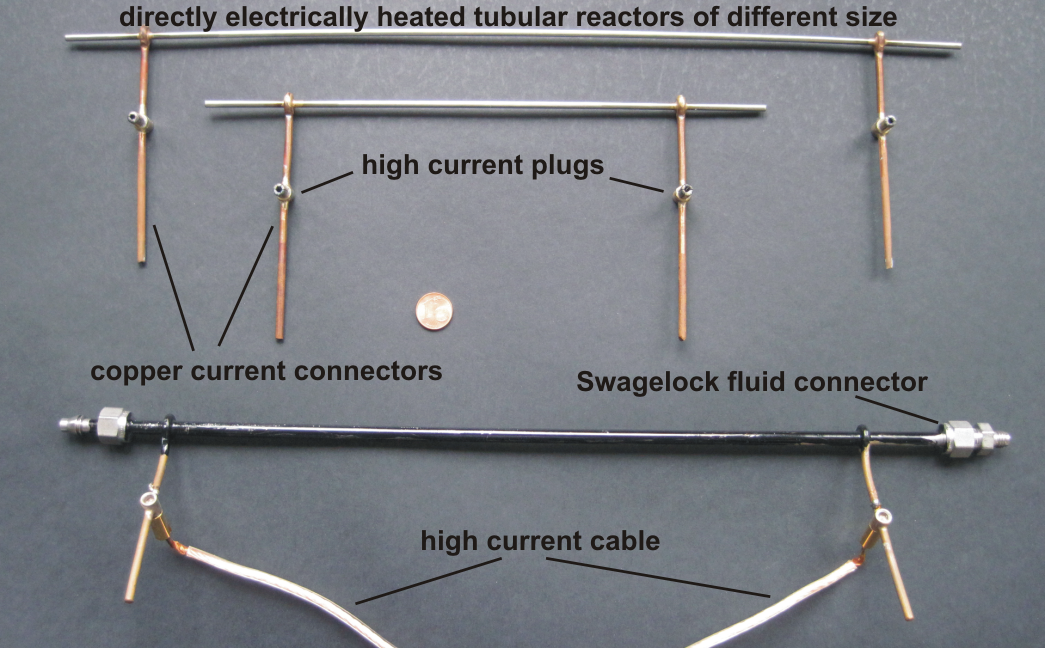

Supplement: File 6 — Photograph of different tubular reactors. [file Beilstein_J_Org_Chem-05-70-s006.tif]
